# Are Malaysian Type 2 Diabetes patients willing to be trained to speak to their offspring about risk of diabetes and preventive measures?

**DOI:** 10.1186/s12875-020-01121-0

**Published:** 2020-03-11

**Authors:** Siti Fatimah Badlishah-Sham, Anis Safura Ramli, Mohamad Rodi Isa, Nurzakiah Mohd-Zaki, David Leonard Whitford

**Affiliations:** 1 Department of Primary Care Medicine, Faculty of Medicine, Universiti Teknologi MARA (UiTM), Selayang Campus, Jalan Prima Selayang 7, 68100 Batu Caves, Selangor Malaysia; 2 Institute of Pathology, Laboratory and Forensic Medicine (I-PPerForM), Universiti Teknologi MARA (UiTM), Sungai Buloh Campus, Jalan Hospital, 47000 Sungai Buloh, Selangor Malaysia; 3 Department of Public Health Medicine, Faculty of Medicine, Universiti Teknologi MARA, Sungai Buloh Campus, Jalan Hospital, 47000 Sungai Buloh, Selangor Malaysia; 4 Department of Medicine, Hospital Kuala Lumpur, Ministry of Health, Jalan Pahang, 50586 Wilayah Persekutuan Kuala Lumpur, Malaysia; 5 RCSI & UCD Malaysia Campus, 4, Jalan Sepoy Lines, 10450 George Town, Pulau Pinang Malaysia

**Keywords:** Diabetes mellitus, offspring, risk perception, training, primary care, Malaysia

## Abstract

**Background:**

Offspring of type 2 diabetes patients have an absolute risk of 20–40% of developing the condition. Type 2 diabetes patients should be encouraged to speak to their offspring regarding diabetes risk and prevention strategies. The Health Belief Model conceptualises that the higher the perceived risk, the more likely an individual will modify their behaviour. The objectives of this study were to i) determine the distribution of type 2 diabetes patients regarding their willingness to accept training to speak to their offspring, ii) determine the distribution of type 2 diabetes patients regarding their willingness to accept training based on the HBM and iii) to determine the factors associated with their willingness to accept training.

**Methods:**

This was a cross-sectional study amongst type 2 diabetes patients attending two primary care clinics in Malaysia. Sociodemographic data and knowledge of diabetes risk factors were collected. The adapted, translated and validated Diabetes Mellitus in the Offspring Questionnaire-Malay version (DMOQ-Malay) was self-administered. Statistical analysis included descriptive statistics, univariate and multiple logistic regression (MLogR).

**Results:**

A total of 425 participants were recruited. Of these, 61.6% were willing to accept training. In MLogR, six variables were found to be significantly associated with willingness to accept training. These were i) positive family history [Adj. OR 2.06 (95% CI: 1.27, 3.35)], ii) having the correct knowledge that being overweight is a risk factor [Adj. OR 1.49 (95%CI: 1.01, 2.29)], iii) correctly identifying age ≥ 40 years old as a risk factor [Adj. OR 1.88 (95%CI: 1.22, 2.90)], iv) agreeing that speaking to their offspring would help them to prevent type 2 diabetes [Adj. OR 4.34 (95%: 1.07, 17.73)], v) being neutral with the statement ‘I do not have much contact with my offspring’ [Adj. OR: 0.31 (95% CI: 0.12, 0.810] and vi) being neutral with the statement ‘my offspring are not open to advice from me’ [Adj. OR: 0.63 (95% CI: 0.31, 0.84].

**Conclusion:**

The majority of type 2 diabetes patients were willing to accept training to speak to their offspring to prevent diabetes. A training module should be designed to enhance their knowledge, attitude and skills to become family health educators.

## Background

Type 2 diabetes is one of the commonest non-communicable diseases (NCD) in Malaysia and its prevalence is rising at an alarming rate. The overall prevalence of type 2 diabetes among adults of ≥18 years old was reported as 17.5% in the latest National Health Morbidity Survey in 2015 [1]. This has shown an increase from 15.2% compared to the previous national survey in 2011 [2]. This clearly demonstrates the importance of diabetes prevention, especially in high-risk groups. One of the high-risk groups of interest is offspring of individuals with type 2 diabetes [3].

Evidence has shown that offspring who have one parent with type 2 diabetes have an absolute risk of 20–40% of developing the condition [4]. Genetic predisposition of an individual is considered an essential factor in the development of type 2 diabetes, but the presence of environmental and behavioural factors further play a role in the activation of these genes [5]. Studies have also shown that family members living together have a predisposition to developing similar diseases as they tend to adopt similar lifestyle behaviours [6]. This demonstrates the pivotal role of lifestyle modification among family members of individuals with type 2 diabetes in order to prevent diabetes [7].

A starting point may be to encourage type 2 diabetes patients to become the promoter of health within their family by talking to their offspring about risk of diabetes [8]. This would be more effective if they were able to promote preventive lifestyle changes as a means to prevent type 2 diabetes in their offspring. However, implementing diabetes prevention strategies and interventions in the family is challenging and less likely to be successful should they not perceive their family members to be at risk of diabetes [9].

Risk perception also known as perceived risk has been extensively studied and forms a central construct of many health behaviour models that addresses health-protective behaviours [10]. The Health Belief Model (HBM) conceptualises that the higher the perceived risk of developing a certain disease, the more likely an individual will modify their behaviour. In the context of diabetes prevention in the offspring, it is hypothesized that type 2 diabetes patients who perceive their offspring to be at risk of developing the condition will be more likely to introduce change within their family as a means of prevention.

Hence, establishing the risk perception of type 2 diabetes patients who have offspring is important prior to introducing preventive lifestyle intervention within their family. This step is crucial to identify type 2 diabetes patients who are willing to motivate and speak to their offspring about adopting risk-reducing behaviour and accept diabetes prevention strategies [11]. Several studies have assessed perceived diabetes risk and the possibility of prevention in the type 2 diabetes population and their offspring [12–15]. Other studies have further investigated the willingness of type 2 diabetes patients to participate in diabetes prevention strategies [8, 12, 16].

Whitford et al. studied the perceived diabetes risk and the willingness of type 2 diabetes patients to speak to their offspring and siblings among the Irish population [8]. They developed a questionnaire in the English language based on the domains of the HBM [17] including knowledge of diabetes risk factors, perceived susceptibility, perceived benefits, perceived barriers and perceived severity. This questionnaire was later named the Diabetes Mellitus in the Offspring Questionnaire (DMOQ) which was adapted, translated, and validated into the Malay language (DMOQ Malay) [18].

However, to date, perceived diabetes risk among type 2 diabetes patients has not been studied in the Malaysian context. This paucity of evidence led to this study which aims to i) determine the distribution of type 2 diabetes patients according to their willingness to accept training to speak to their offspring, ii) determine the distribution of type 2 diabetes patients according to their willingness to accept training based on the domains of the HBM and iii) to determine the factors associated with type 2 diabetes patients’ willingness to accept training.

## Methods

### Study design and setting

This was a cross-sectional study carried out in two primary care clinics in the state of Selangor, Malaysia between July to August 2016. One of the clinics was located in a semi urban area while the other clinic was located in an urban area. The two centres provided a good diversity of racial backgrounds of patients.

### Study population

The participants recruited for this study were type 2 diabetes patients who were followed up at the two primary care clinics. The inclusion criteria included type 2 diabetes patients who were ≥ 18 years old, had at least one offspring without type 2 diabetes and were able to speak and understand the Malay language. Patients were excluded if they had type 1 diabetes, were pregnant, had gestational diabetes, had a previous or current history of mental disorders, had visual impairment that may impede the administration of the study tool or could not speak or understand the Malay language.

### Sampling method

Type 2 diabetes patients attending the clinics were approached consecutively during the data collection days, given a patient information sheet describing the study and were invited to participate. Patients who agreed were then screened to assess whether they met the inclusion and exclusion criteria. Medical records were also checked for secondary data for confirmation of details. Those who were eligible were recruited into the study and written informed consent was obtained.

### Study tool

The tool that was used in this study was the DMOQ Malay version [18]. This self-administered questionnaire was used to assess the perceptions of type 2 diabetes patients towards their offspring’s risk of developing type 2 diabetes and the possibility of prevention. The English version of this questionnaire was originally developed in 2009 by Whitford et al. [8] based on the domains of the HBM which includes perceived susceptibility, perceived benefits, perceived severity and perceived barriers [17]. It was later adapted, translated and validated into the Malay language [18]. The DMOQ Malay version comprised of 21 items framed within five domains: 1) knowledge of type 2 diabetes risk factors, 2) perceived susceptibility, 3) perceived benefits, 4) perceived barriers and 5) perceived severity. The Cronbach alpha was 0.714 and the intraclass-correlation coefficient was > 0.7 [18].

### Data collection and study procedures

Data was collected by a research assistant (RA) who was trained with regards to the study procedures to minimize variability in the method of data collection. Socio-demographic characteristics were collected via face-to-face interview of the participants which includes their age, gender, ethnicity, family history of type 2 diabetes, number of children without type 2 diabetes, personal status and the highest formal education. These details were recorded in a standardised case report form (CRF) along with data from the medical records of participants which were obtained for the purpose of confirming the duration of type 2 diabetes and the current treatment for type 2 diabetes.

### Administration of questionnaire

Participants were given the DMOQ Malay version with clear instructions on how to fill in the questionnaire. They were asked to circle the options that suited them the most as well as to answer the subjective questions in the space given. Participants were advised to seek for clarification from the RA should any queries arise. They were advised to answer the questionnaires themselves. Most of the participants took approximately 10 to 15 min to complete the questionnaire. Once the questionnaire was completed, it was handed to the RA and checked for completeness.

### Sample size calculation

The sample size was calculated using the single proportion formula with 5% precision and 95% confidence interval. The proportion (P) was estimated based on a study by Whitford et al., which showed that 56% of type 2 diabetes patients would speak to family members about their risk of developing diabetes if they were offered training to do so [8]. The calculated required sample size was 379. Taking into consideration an additional 20% of participant refusal and non-eligibility rate, this study aimed to approach 455 patients.

### Statistical analysis

The data in this study was analysed using the Statistical Package for Social Sciences (SPSS) version 22.0 (IBM). Variables were described as mean ± standard deviation (±SD) for continuous data and number (n) and percentage (%) for dichotomous or nominal data. The scores for items 1 to 6 in section 5 of the DMOQ Malay version were reversed as the questions in this section were negatively phrased. The factors associated with willingness of type 2 diabetes patients to accept training to speak to offspring were analysed by simple logistic regression (SLogR) followed by multiple logistic regression (MLogR) as the data consisted of categorical variables. Sociodemographic characteristics, knowledge of risk factors of type 2 diabetes and items of all the factors of the HBM from the DMOQ were the independent variables entered into the SLogR. Variables with a *p*-value of less than 0.05 from the SLogR were then included in the MLogR analysis. A *p*-value of less than 0.05 was considered statistically significant in the MLogR.

## Results

**Fig. 1:**
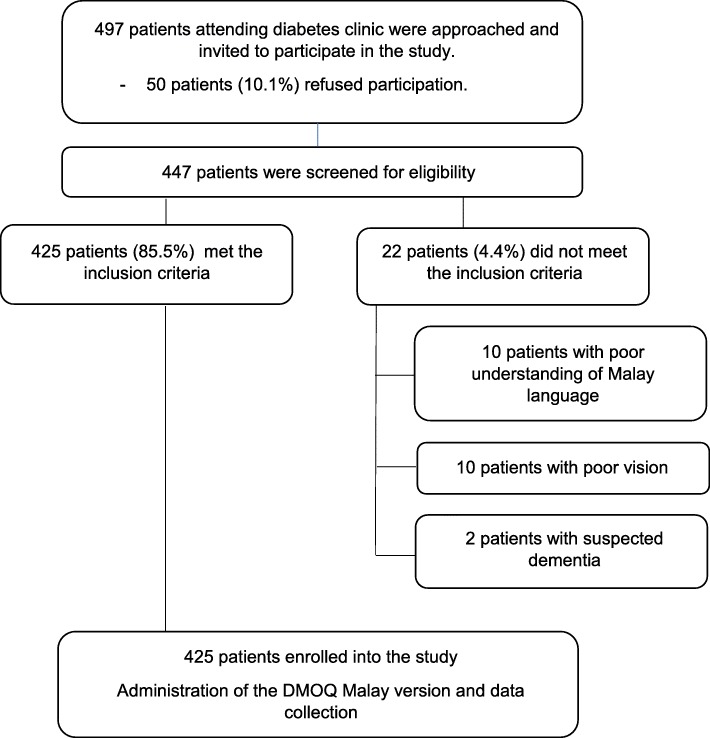
Flow chart of patient recruitment

Figure 1 shows the flow chart of patient recruitment. A total of 497 type 2 diabetes patients were invited to enter the study. Out of this, 50 patients (10.1%) refused to participate. Therefore, 447 patients were screened for eligibility and 22 patients (4.4%) did not fulfil the eligibility criteria. Consequently, 425 patients who met the eligibility criteria were recruited into the study giving a recruitment rate of 85.5%.

**Fig. 2:**
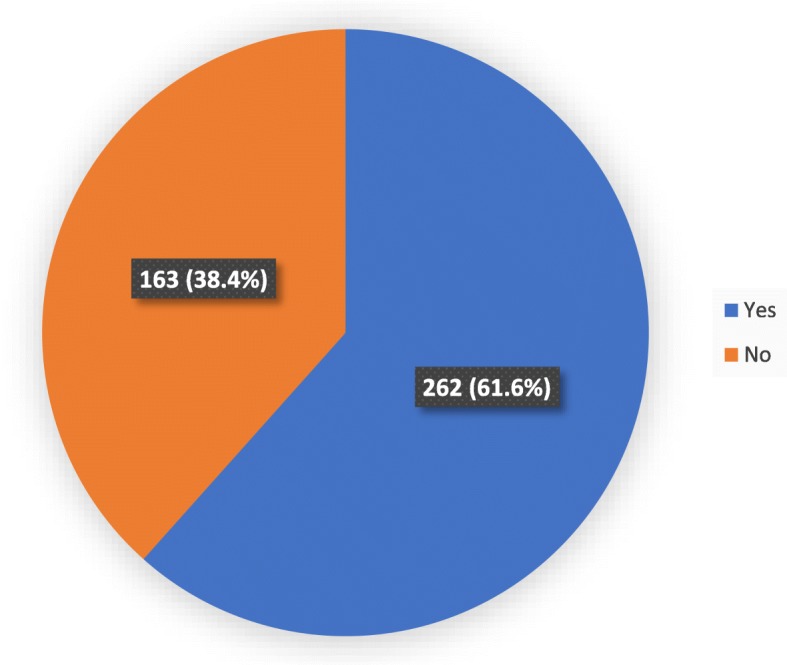
Distribution of type 2 diabetes patients according to their willingness to accept training to speak to their offspring (*N* = 425)

Figure 2 shows the distribution of type 2 diabetes patients according to their willingness to accept training to speak to their offspring. Out of 425 participants, 61.6% of them were willing to accept training.

The demographic characteristics of the participants are shown in Table 1. The proportion of participants with a family history of type 2 diabetes who were willing to accept training was higher (80.2%) compared to those who were not willing (69.3%). Otherwise, the demographic characteristics were comparable between those who were willing to accept training and those who were not.

**Table 1:** Demographic characteristics of the participants

Variables	Willing to accept training		Total (***N*** = 425), n(%)
	Yes (***N*** = 262), n(%)	No (***N*** = 163), n(%)	
**Age** (years old):			
[Mean (SD)]	54.33 (8.39)	56.05 (8.76)	54.99 (8.57)
18–29	1 (0.4)	2 (1.2)	3 (0.7)
30–59	184 (70.2)	94 (57.7)	278 (65.4)
60 and above	77 (29.4)	67 (41.1)	144 (33.9)
**Gender:**			
Male	131 (50.0)	77 (47.2)	208 (48.9)
Female	131 (50.0)	86 (52.8)	217 (51.1)
**Ethnicity:**			
Malay	230 (87.8)	143 (87.7)	373 (87.8)
Chinese	8 (3.1)	8 (4.9)	16 (3.8)
Indian	17 (6.5)	9 (5.5)	26 (6.1)
Bumiputera (Sabah & Sarawak)	3 (1.1)	1 (0.6)	4 (0.9)
Others	4 (1.5)	2 (1.0)	6 (1.4)
**Marital status:**			
Married	234 (89.3)	137 (84.0)	371 (87.8)
Widowed	23 (8.8)	22 (12.5)	45 (10.6)
Divorce	4 (1.5)	3 (1.8)	7 (1.6)
Not married	1 (0.6)	1 (0.6)	2 (0.5)
**Education:**			
No	5 (1.9)	5 (3.1)	10 (2.4)
Primary	35 (13.4)	29 (17.8)	64 (15.1)
Secondary	145 (55.3)	90 (55.2)	235 (55.3)
Tertiary	77 (29.4)	39 (23.9)	116 (27.3)
**Duration of type 2 diabetes (Years):**			
[Mean (SD)]	7.32 (5.91)	8.05 (6.95)	7.60 (6.33)
Less than 5 years	111 (42.4)	66 (40.5)	177 (41.6)
5–10 years	92 (35.1)	60 (36.8)	152 (35.8)
10 years and above	59 (22.5)	37 (22.7)	96 (22.6)
**Treatment:**			
Diet only	9 (3.4)	6 (3.7)	15 (3.5)
Oral antidiabetic & Diet	165 (63.0)	103 (63.2)	268 (63.1)
Diet & Insulin	14 (5.3)	13 (8.0)	27 (6.4)
Oral antidiabetic, diet & insulin	74 (28.2)	41 (25.2)	115 (27.1)
**Family history of type 2 diabetes:**			
Yes	210 (80.2)	113 (69.3)	323 (76.0)
No	52 (19.8)	50 (30.7)	102 (24.0)
**No. of offspring without type 2 diabetes:**			
1–3	138 (52.7)	80 (49.1)	218 (51.3)
4 and above	124 (47.3)	83 (50.9)	207 (48.7)

Table 2 shows the distribution of type 2 diabetes patients according to their willingness to accept training based on the domains of the HBM. For perceived susceptibility, two items were found to have significant trends which were ‘likelihood that their offspring is likely to get diabetes’ (χ^2^ = 6.760, 2 d.f.; *p* = 0.034) and ‘worry that their offspring will get diabetes’ (χ^2^ = 11.196, 2 d.f.; *p* = 0.004). In the perceived benefits, there were also two items found to have significant trends which were ‘talking to their offspring would make them more aware of importance of diet and exercise’ (χ^2^ = 6.535, 2 d.f.; *p* = 0.038) and ‘encourage their offspring to make lifestyle changes’ (χ^2^ = 16.652, 2 d.f.; *p* < 0.001). Two items from the domain of perceived barriers were found to show significant trends which were ‘I do not have much contact with my offspring’ (χ^2^ = 12.892, 2 d.f.; *p* = 0.002) and ‘my offspring are not open to advice from me’ (χ^2^ = 8.843, 2 d.f.; *p* = 0.012). There is no item in perceived severity found to be significant.

**Table 2:** Distribution of type 2 diabetes patients according to their willingness to accept training based on the domains of the Health Belief Model

Domains of the Health Belief Model	Willing to accept training		***x***^***2***^ (df)^**a**^	***p***-value
	Yes (***N*** = 262) n (%)	No (***N*** = 163) n (%)		
**PERCEIVED SUSCEPTIBILITY:**				
**Likelihood that offspring will get diabetes:**				
Not likely	43 (16.4)	28 (17.2)	6.760	0.034*
Neutral	39 (14.9)	40 (24.5)	(2)	
Likely	180 (68.7)	95 (58.3)		
**Likelihood someone without family history of diabetes will get diabetes:**				
Not likely	33 (12.6)	14 (8.6)	5.518	0.063
Neutral	10 (3.8)	14 (8.6)	(2)	
Likely	219 (83.6)	135 (82.8)		
**Worry that offspring will get diabetes:**				
Not worry	37 (14.1)	28 (17.2)	11.196	0.004*
Neutral	11 (4.2)	20 (12.3)	(2)	
Worry	214 (81.7)	115 (70.5)		
**PERCEIVED BENEFITS:**				
**Talking make offspring more aware of importance of diet and exercise:**				
Disagree	3 (1.1)	6 (3.7)	6.535	0.038*
Neutral	2 (0.8)	5 (3.1)	(2)	
Agree	257 (98.1)	152 (93.2)		
**Encourage offspring to make lifestyle changes:**				
Disagree	3 (1.1)	8 (4.9)	16.652	< 0.001*
Neutral	2 (0.8)	10 (6.1)	(2)	
Agree	257 (98.1)	145 (89.0)		
**Help prevent type 2 diabetes:**				
Disagree	5 (1.9)	10 (6.1)	5.589	
Neutral	6 (2.3)	5 (3.1)	(2)	
Agree	251 (95.8)	148 (90.8)		0.061
**PERCEIVED BARRIERS**				
**I do not have a healthy lifestyle myself:**				
Agree	108 (41.2)	62 (38.0)	2.204	0.322
Neutral	33 (12.6)	29 (17.8)	(2)	
Disagree	121 (46.2)	72 (44.2)		
**I do not have much contact with my offspring:**				
Agree	45 (17.2)	20 (12.3)	12.892	0.002*
Neutral	13 (5.0)	24 (14.7)	(2)	
Disagree	204 (77.8)	119 (73.0)		
**My offspring are not open to advice from me:**				
Agree	54 (20.6)	30 (18.4)	8.843	0.012*
Neutral	36 (13.7)	41 (25.1)	(2)	
Disagree	172 (65.7)	92 (56.5)		
**They do not see diabetes as a serious illness:**				
Agree	79 (30.2)	46 (28.2)	0.844	0.656
Neutral	25 (9.5)	20 (12.3)	(2)	
Disagree	158 (60.3)	97 (59.5)		
**They do not believe they are at risk for diabetes:**				
Agree	75 (28.6)	42 (25.8)	3.496	0.174
Neutral	42 (16.1)	38 (23.3)	(2)	
Disagree	145 (55.3)	83 (50.9)		
**I prioritize other things than my own health:**				
Agree	197 (75.2)	120 (73.6)	0.132	0.936
Neutral	24 (9.2)	16 (9.8)	(2)	
Disagree	41 (15.6)	27 (16.6)		
**PERCEIVED SEVERITY**				
	**Mean** **(95%CI)**	**Mean** **(95%CI)**	**t (df)**^b^	***p*-value**
Cancer	4.41 (4.30, 4.52)	4.33 (4.19, 4.48)	0.81 (423)	0.075
DM	4.32 (4.21, 4.42)	4.17 (4.02, 4.31)	1.723 (422)	0.086
AIDS	4.39 (4.23, 4.51)	4.23 (4.04, 4.42)	1.22 (423)	0.154

Notes:

* Statistically significant at α = 0.05

^a^Statistical test: Chi-square

^b^Statistical test: Student t-test

Eleven significant variables from SLogR were included into the MLogR analysis. These include age group (*p* = 0.025); family history of type 2 diabetes (*p* = 0.015); knowledge of type 2 diabetes risk factors which were overweight (*p* = 0.038); and age more than 40 years old (*p* = 0.012), ‘likelihood that offspring will get diabetes’ (*p* = 0.036),‘likelihood someone without family history of type 2 diabetes will get type 2 diabetes (neutral vs not likely, *p* = 0.022), ‘worry that offspring will get diabetes’ (*p* = 0.006), ‘encourage offspring to make lifestyle changes’ (*p* = 0.002), ‘help prevent type 2 diabetes (agree vs disagree, *p* =0.028), ‘I do not have much contact with my offspring’ (*p* =0.003) and ‘my offspring are not open to advice from me’ (*p* = 0.013).

Table 3 shows the factors associated with willingness of type 2 diabetes patients to accept training to speak to their offspring. In MLogR, six variables were found to be significantly associated with willingness of type 2 diabetes patients to accept training to speak to their offspring. These included family history of type 2 diabetes [Adj. OR 2.06 (95% CI: 1.27, 3.35)], knowledge of overweight as a risk factor for type 2 diabetes [Adj. OR 1.49 (95%CI: 1.01, 2.29)], knowledge of age ≥ 40 years old as a risk factor for type 2 diabetes [Adj. OR 1.88 (95%CI: 1.22, 2.90)], perceived benefit of speaking to offspring would help prevent type 2 diabetes [Adj. OR 4.34 (95% CI: 1.07, 17.73)], participants who were neutral with the statements ‘I do not have much contact with my offspring’ [Adj. OR: 0.31 (95% CI: 0.12, 0.810] and ‘my offspring are not open to advice from me’ [Adj. OR: 0.63 (95% CI: 0.31, 0.84].

**Table 3:** Factors associated with willingness of type 2 diabetes patients to accept training to speak to their offspring regarding risks of type 2 diabetes and means of prevention

Variables	Multiple Logistics Regression (MLogR)			
	Adj. Beta (SE)	Wald (df)	***p***-value	Adj. OR (95%CI)
**DEMOGRAPHIC CHARACTERISTICS**				
**Family history of type 2 diabetes:**				
Yes	0.72 (0.25)	8.564 (1)	0.003*	2.06 (1.27, 3.35)
No				1
**KNOWLEDGE OF RISK FACTORS**				
**Overweight:**				
Yes	0.40 (0.22)	3.843	0.045*	1.49 (1.01, 2.29)
No				1
**Age more than 40:**				
Yes	0.63 (0.22)	8.280 (1)	0.04*	1,88 (1.22, 2.90)
No				1
**PERCEIVED BENEFIT**				
**Speaking to offspring helps them to prevent diabetes:**				
Disagree		9.537 (2)	0.008	1
Neutral	−0.42 (1.08)	0.153 (1)	0.696	0.66 (0.80, 5.40)
Agree	1.47 (0.72)	4.242 (1)	0.039*	4.34 (1.07, 17.73)
**PERCEIVED BARRIER**				
**I do not have much contact with my offspring:**				
Agree		5.988 (2)	0.005	1
Neutral	−1.16 (0.49)	5.718 (1)	0.017*	0.31 (0.12, 0.81)
Disagree	−0.33 (0.34)	0.969 (1)	0.325	0.72 (0.37, 1.39)
**My offspring are not open to advice from me:**				
Agree		5.528 (2)	0.063	1
Neutral	−0.46 (0.36)	1.641 (1)	0.045*	0.63 (0.31, 0.84)
Disagree	0.21 (0.31)	0.439 (1)	0.508	1.23 (0.67, 2.27)

Notes:

Hosmer and Lemeshow test =0.849

Variables with a *p-*value of < 0.05 with simple logistic regression were included in the multiple logistic regression

Multiple logistic regression (no multicollinearity)

All assumptions were met

Sensitivity: 88.9%, specificity: 29.4%

*p-*value = *p-*value from Wald’s tests

*CI* Confidence interval, *df* Degree of freedom, *OR* Odds ratio

* Statistically significant at *p* = 0.05

## Discussion

### Main findings of study and comparison with previous literature

This was the first study in Malaysia determining the distribution of type 2 diabetes patients who were willing to accept training to speak to their offspring to prevent diabetes and the factors associated with it. Our study shows that 61.6% were willing to accept training to speak to their offspring, a figure comparable to that (56%) from a previous study in Ireland [8]. A subsequent study conducted by the same group of researchers comparing type 2 diabetes patients in Ireland and Bahrain showed that the proportion of patients willing to speak to their family members was significantly higher in Ireland compared to Bahrain (75% vs. 54%, *p* < 0.001) [12]. These findings suggest that type 2 diabetes patients in these countries are willing to accept training if offered. This opportunity should be explored further and a training module for type 2 diabetes patients should be developed as a potential means of preventing diabetes in their offspring. At present, the evidence on effectiveness of this intervention is lacking. A randomised controlled trial is required to prove its value.

In the multivariate analysis, six variables were found to be significantly associated with the willingness of type 2 diabetes patients to accept training to speak to their offspring. These are i) having a family history of type 2 diabetes, ii) correctly identifying that overweight is a diabetes risk factor, iii) correctly identifying age ≥ 40 years old as a diabetes risk factor, iv) perceiving the benefit of speaking to offspring to help prevent them from developing diabetes, v) perceiving not having much contact with offspring as a barrier and vi) perceiving their offspring to not being open to advice from them as a barrier.

Type 2 diabetes patients who have a positive family history were twice as likely to be willing to accept training compared to those who did not have a family history [Adj. OR 2.06 (95% CI: 1.27, 3.35)]. Direct comparison to other studies is not possible as no data was presented in the same manner. Our finding is unique and highlights the importance of targeting those with a strong family history of diabetes in our population in terms of training them to speak to their offspring to prevent diabetes.

With regards to knowledge of risk factors, participants who had the correct knowledge that being overweight [Adj. OR 1.49 (95%CI: 1.01, 2.29)] and age ≥ 40 years old [Adj. OR 1.88 (95%CI: 1.22, 2.90)] are risk factors for type 2 diabetes were more likely to be willing to accept training compared to those who did not know. Again, direct comparison to other studies is not possible as no data was presented in similar manner. Our study shows that enhancing knowledge of type 2 diabetes risk factors among patients would potentially improve their willingness to accept training for diabetes prevention in their offspring.

In terms of perceived susceptibility, our multivariate analysis did not reveal that these items were significantly associated with willingness to accept training. Direct comparison with other studies was not possible as no data was presented in a similar presentation. However, Whitford *et. al.* found that Irish type 2 diabetes patients who worried about their children developing diabetes were more likely to speak to their family members about their risk of diabetes [OR 4.37 (95% CI: 1.75, 10.92)] [8].

Regarding perceived benefits, patients who agreed that speaking to their offspring would help them to prevent type 2 diabetes, were four times more likely to be willing to accept training compared to those who disagreed [Adj. OR 4.34 (95%: 1.07, 17.73)]. This is consistent with the study by Whitford *et. al.* which showed that patients who exhibited an increased appreciation of the benefits of speaking to their offspring were more likely to have engaged in preventive behaviours [8]. Perceived benefit is reflected as the individual’s estimate of a likelihood that a given action will achieve a specific goal [17]. However, in the context of preventing diabetes, the challenge would be to educate those who do not appreciate the importance of speaking to their offspring.

In terms of perceived barriers, patients who were neutral with the statements ‘I do not have much contact with my offspring’ [Adj. OR: 0.31 (95% CI: 0.12, 0.810] and ‘my offspring are not open to advice from me’ [Adj. OR: 0.63 (95% CI: 0.31, 0.84], were more likely to be willing to accept training compared to those who agreed with the negative statements. This is comparable to a study by Becker *et. al.* which found that ‘perceived barriers’ construct of the HBM to be the most powerful construct across various preventive health study designs and behaviour [17]. However, our findings are unique as patients who were neutral with the statements on communication with their offspring are more likely to be willing to accept training.

Our study therefore suggests that emphasizing HBM parameters when consulting type 2 diabetes patients in the clinical setting may lead to an increased willingness to accept training to initiate discussion with their offspring.

### Strengths and limitations of the study

The main strength of this study was that it revealed the willingness of type 2 diabetes patients to accept training to speak to their offspring and the factors associated with it. Additionally, the study utilised the DMOQ Malay version which is a valid and reliable tool based on the constructs of HBM to assess the perceptions of type 2 diabetes patients towards their offspring’s risk of developing type 2 diabetes and the possibility of prevention. This study has several limitations. Majority of the patients who were included in this study were of the Malay ethnic group (87.8%) as the DMOQ Malay version could only be administered to participants who were able to read and understand the Malay language. Therefore, the findings of this study may not be generalisable to the Malaysian population which currently consists of the following ethnicities which are Malay (69.3%), Chinese (22.8%), Indian (6.9%) and other ethnicities (1%) [19]. Another limitation was the use of convenience sampling which could have introduced a sampling bias. To minimise this bias, all patients with type 2 diabetes in the waiting area of both study sites were invited to participate in this study during the period of data collection.

### Implications for clinical practice and future research

Findings from this study suggest that type 2 diabetes patients in Malaysia are willing to accept training if offered. A training module should be developed to train type 2 diabetes patients to speak to their offspring as a potential means of preventing diabetes. Due to the potential of social influence within families as shown in this study, interventions should be designed with the goals to enhance knowledge, attitude and skills of type 2 diabetes patients to become family health educators and model healthy behaviours. It should also facilitate intra-familial communication about risk-reducing behaviours. The module should include i) strengthening knowledge on diabetes risk factors, ii) improving attitude and perception towards the benefit of speaking to offspring to help prevent them from developing diabetes and iii) enhancing communication skills to speak to their offspring. Further research should involve other primary care clinics in Malaysia with multi-ethnic background to ensure generalisability of the findings to the Malaysian population. There is also a need for further research to explore the views of perceived diabetes risk in the offspring of type 2 diabetes patients and their willingness to engage in preventive lifestyle behaviour. Future research should include a pragmatic randomised controlled trial to evaluate the effectiveness of the training module.

## Conclusions

This study has shown that a majority of type 2 diabetes patients were willing to accept training to speak to their offspring to prevent diabetes. A training module should be designed with the goals to enhance knowledge, attitude and skills of these patients to become family health educators and model healthy behaviours. The target group should include those with a positive family history of type 2 diabetes. This study should also prompt future research into preventing diabetes among offspring of type 2 diabetes patients in Malaysia.

## Data Availability

Data of this study is kept at the Institute of Pathology, Laboratory and Forensic Medicine (I-PPerForM), Universiti Teknologi MARA (UITM), Sungai Buloh Campus, Jalan Hospital, 47000 Sungai Buloh, Selangor, Malaysia. Data will be shared by the corresponding author upon request and it is subjected to the data protection regulations.

## Abbreviations

Adj OR: Adjusted odds ratio
CRF: Case report form
DMOQ: Diabetes Mellitus in the Offspring Questionnaire
HBM: Health Belief Model
MLogR: Multiple logistic regression
NCD: Non-communicable diseases
RA: Research assistant
SD: Standard Deviation
SLogR: Simple logistic regression
SPSS: Statistical Package for Social Sciences
